# Molecular docking investigation of the amantadine binding to the enzymes upregulated or downregulated in Parkinson’s disease

**DOI:** 10.5599/admet.854

**Published:** 2020-06-15

**Authors:** Mihaela Ileana Ionescu

**Affiliations:** 1 Department of Microbiology, Iuliu Hațieganu University of Medicine and Pharmacy, 6 Louis Pasteur, 400349, Cluj-Napoca, Romania, mionescu@umfcluj.ro; 2 Department of Microbiology, County Emergency Clinical Hospital, 400006, Cluj-Napoca, Romania

**Keywords:** human adenylate kinases, adenine phosphoribosyltransferase, adenine metabolism, purine metabolism

## Abstract

Parkinson’s disease (PD) is a progressive neurodegenerative disease. Levodopa in combination with amantadine has a demonstrated efficacy in motility impairment. An extensive investigation of some enzymes described to be upregulated or downregulated in PD was made – adenylate kinase (AK), adenine phosphoribosyltransferase (APRT), ectonucleoside triphosphate diphosphohydrolase 1 (ENTPD1), nucleoside-diphosphate kinase 3 (NDK3), purine nucleoside phosphorylase 1 (PNP1), and ecto-5’-nucleotidase (NT5E). Also, creatine kinase (CK) was included in the study because it is one of the main enzymes involved in the regulation of the nucleotide ratio in energy metabolism. To date, there is no proven link between amantadine treatment of PD and these enzymes. Because there are many AKs isoforms modified in PD, the AK was the first investigated. The molecular docking experiments allow the analysis of the selective binding of amantadine – unionized (with –NH_2_ group) and ionized form (with –NH_3_^+^ group) – to the AKs’ isoforms implicated in PD. Using available X-ray 3D structures of human AKs in closed-conformation, it was demonstrated that there are notable differences between the interactions of the two forms of amantadine for the zebrafish AK1 (5XZ2), human AK2 (2C9Y), human AK5 (2BWJ), and AK from B.stearothermophilus. The cytosolic human AK1 and human AK2 mostly interact with ionized amantadine by AMP binding residues. The human AK5 interaction with ionized amantadine does not involve the residues from the catalytic site. Among other enzymes tested in the present study, APRT revealed the best results in respect of binding amantadine ionized form. The results offer a new perspective for further investigation of the connections between amantadine treatment of PD and some enzymes involved in purine metabolism.

## Introduction

Parkinson’s disease (PD) is an idiopathic neurodegenerative and progressive disease, which mainly produces motility impairment with bradykinesia, tremor, and postural instability [1, 2]. In PD, the neurotransmitter dopamine is reduced as a result of the degeneration of the dopaminergic neurons from the *substantia nigra* [3]. The PD-related drug utilization and PD-related other degenerative diseases are due to the losses/impairment of the dopamine action at the basal ganglions [4, 5]. Also, some toxins (6-hydroxydopamine) are identified to induce dopaminergic cell death [6–8]. PD is a chronic disease with no curative treatment of the underline disease. Therefore is an ongoing interest in founding the most efficient strategy for treatment the motility disorders and other symptoms [9–14]. The drugs used in the treatment of PD should correct the dopamine/cholinergic balance, because the level of dopamine in the caudate-putamen in the striatum is considerably reduced, with the exacerbation of the cholinergic control [15, 16]. The most used dopaminergic drugs are levodopa, bromocriptine, and amantadine whereas the main anti-cholinergic drug is trihexyphenidyl [17–20].

Levodopa, discovered in 1950, was first approved in PD treatment by the FDA (U.S. Food and Drug Administration) in 1975 in combination with carbidopa, on the product called Sinemet [21]. An important side effect of long-term use of levodopa is levodopa-induced dyskinesia [22]. In this case, amantadine is the only treatment option that has been shown to be effective [23]. According to DrugBank (https://www.drugbank.ca/), amantadine (accession number DB00915) is not only an antiviral used for prophylaxis and treatment in infections with influenza A but, also an effective antiparkinsonian in combination with levodopa. The mechanism of action of amantadine is not entirely understood. According to PubChem (https://pubchem.ncbi.nlm.nih.gov/), amantadine (PubChem CID 2130) stimulates the release of dopamine from striatal dopaminergic nerve terminals and inhibits the pre-synaptic uptake of dopamine.

In neurodegenerative diseases, among other biomarkers, some AKs isoforms are dysregulated. There is demonstrated that in stages 3-6 of the PD there is a down-regulation of the AK2, AK3 and AK4 in the *substantia nigra*. Also, there is a reduced expression of adenine phosphoribosyltransferase (APRT), ectonucleoside triphosphate diphosphorylase 1 (ENTPD1), nucleoside-diphosphate kinase 3 (NDK3) and purine nucleosise phosphorylase 1 (PNP1) mRNA. On contrary, the stages 5-6 of PD are characterized by the up-regulation of the AK1, AK5, nucleoside-diphosphate kinase 5 (NDK5), ecto-5’-nucleotidase (NT5E) and purine nucleoside phosphorylase 1(PNP1) [24]. As the changes in the purine metabolism influence the evolution of PD and, ultimately, the effectiveness of the treatment, the study of human AKs isoforms and enzymes involved in the purine metabolism is of great interest.

The present study aimed to analyze the connection between the dysregulated enzymes in different stages of PD and amantadine. It is noteworthy that different AKs isoforms are modified in PD. The elements that link the AK1 and AK2 isoforms and amantadine are dyskinesia and inflammation. AK1 and AK2 isoforms are altered in other muscle diseases – Duchenne muscular dystrophy – or in intense physical activity [25–30]. It has long been known, on the one hand, the positive role or amantadine in managing dyskinesia from PD and, on the other hand, the AK1 and AK2 dysregulation in PD. However, to date, there is no proven link between amantadine and AK in PD. Before planning a strategy that implies costly and time-consuming experiments, the molecular docking experiments were chosen to perform the present study.

The AK was the first enzyme analyzed because the specific conformational movements of the AKs during catalysis are well studied and there are many X-ray 3D structures of these conformations of the AKs deposed in the Protein Data Bank (PDB). Therefore, molecular docking, despite the limitations of the method, allows a first look at the connection between PD, amantadine, and some enzymes already demonstrated to be dysregulated in PD. We analyzed the amantadine ionized form (with –NH_3_^+^ group) because the amantadine is ionized in physiological conditions [31].

To verify the hypothesis of that amantadine specifically target some enzymes modified in PD, the following enzymes were analyzed – adenylate kinase (AK), adenine phosphoribosyltransferase (APRT), ectonucleoside triphosphate diphosphohydrolase 1 (ENTPD1), nucleoside-diphosphate kinase 3 (NDK3), purine nucleoside phosphorylase 1 (PNP1), and ecto-5’-nucleotidase (NT5E). More, because in skeletal muscle AK1 and creatinine kinase (CK) are the main enzymes involved in the regulation of nucleotide ratio in energy metabolism, the molecular docking of CK was an additional analysis [27].

## Experimental

The study workflow is as follows: (1) retrieving and analysis of the enzymes with X-ray 3D crystalline structure deposed in public databases, (2) molecular docking procedure with amantadine ionized form (with –NH_3_^+^ group), (3) redocking of the substrates/substrates analogs with AKs, (4) multiple sequences alignment of AKs, (5) retrieving and analysis of the APRT, ENTPD1, NDK3, PNP1, NT5E, and CK with X-ray 3D crystalline structure deposed in public databases, (6) molecular docking procedure with amantadine ionized form (with –NH_3_^+^ group) of the following enzymes: APRT, ENTPD1, NDK3, PNP1, NT5E, and CK.

### Adenylate kinase (AK)

The human AKs isoforms and selected other AKs from other organisms were retrieved from the PDB (https://www.rcsb.org/). The characteristics of the AKs tested (PDB ID(s) 2C95, 1Z83, 5XZ2, 2C9Y, 2AR7, 2BBW, 2BWJ, 3HPQ, and 1ZIP) are synthesized in Table S1 [32–34].

### Human adenine phosphoribosyltransferase (APRT)

The human APTRs co-crystallized with the substrate AMP and with adenine, allow a deep view of the amantadine interactions with the residues involved in catalysis The X-ray crystal structures of the APTRs included in the study (PDB ID(s) 1ORE, 1ZN7, 1ZN8, 1ZN9, 4X44, and 6FCI) are presented in the Table S2 [35–37].

### Human ecto-5’-nucleotidase (NT5E)

The human NT5Es co-crystallized with the substrate adenosine and with phosphomethylphosphonic acid adenosyl ester, allow a deep view of the amantadine interactions with the residues involved in catalysis. The X-ray crystal structures of the NT5Es included in the study (PDB ID(s) 4H2I, 4H2G, 4H2F, and 4H1S) are presented in the Table S3 [38, 39].

### Human ectonucleoside triphosphate diphosphohydrolase 1 (ENTPD1)

The human NDK3s in closed-conformation and without ligands allows a thorough analysis of the amantadine interactions with the catalytic site. The X-ray crystal structures of the NDK3s included in the study (PDB ID(s) 1ZS6, 2HVD, 2HVE, 1JXV, and 3BBB) are presented in the Table S4 [40–42].

### Human purine nucleoside phosphorylase 1 (PNP1)

The many X-ray 3D structures of human PNP1 co-crystallized with the substrate or analag substrate allows a good interpretation of the molecular docking results of amantadine with PNP1. The X-ray crystal structures of the PNP1s included in the study (PDB ID(s) 1ULA, 1ULB, 2A0W, 2A0X, 2A0Y, 1RSZ, and 1RFG) are presented in the Table S5 [43–45].

### Creatinin kinases (CK)

Due to the different isoforms of the CKs, all these isoforms were selected. X-ray 3D structures of human CKs are compared to CKs of other organisms, due to the small number of human CKs structures deposed in PDB. The X-ray crystal structures of the CKs included in the study (PDB ID(s) 1CRK, 4Z9M, 2CRK, 1I0E, 1U6R, 3DRB, 3DRE, 1QH4, and 1G0W) are presented in the Table S6 [46–52].

### Inclusion and exclusion criteria

Inclusion criteria: The human AKs and AKs form other eucaryotes with X-ray 3D structure in closed-conformation – co-crystallized with substrate or analog substrate. For further comparison, AK1 from zebrafish (*Danio rerio*) and two bacterial AKs in closed-conformation were included – one from a Gram-negative bacteria, *E.coli*, and one from a Gram-positive bacteria, *B.stearothermophilus*. Other human enzymes described to be dysregulated in PD (APRT, NT5E, ENTPD1, NDK3, and PNP1). The human CKs were also tested because their major role in nucleotide ratios in muscles. When enzyme of human origin was not found in PDB, the same enzyme of other origin was retrieved.

Exclusion criteria: Human AKs in open conformation or with no X-ray 3D structure records in public databases. AKs from other organisms with the exception of the AKs selected in the inclusion criteria for further comparisons.

### Molecular docking procedure

#### Adenylate kinases preparation as receptors

For the present study were selected from PDB Database (https://www.rcsb.org/) the AKs 3D structures in closed conformation – co-crystallized with substrate or analog substrate. The AKs X-ray 3D structures downloaded from the PDB Database as pdb files were prepared as receptors. All solvent molecules, the ions and the substrates were removed. In the case of multimeric AKs, only the chain that was crystallized with substrate or substrate analog was selected. Three chains were selected for human AK5 2BWJ – chains B, C, and F – all three crystallized with AMP but with different interactions with AMP. Thus, in 3D crystal structure from PDB, two residues in the chain B form hydrophobic interaction with AMP (Gln29 and His39) and the Gln29 in the chain F forms a hydrophobic interaction with AMP and no interaction with AMP for chain C.

#### Ligand preparation

The 3D structure of ligands was retrieved from the PubChem Compound Database (https://www.ncbi.nlm.nih.gov/pccompound/). Molecular docking experiments were performed with the unionized amantadine (PubChem CID 2130) and ionized amantadine (with –NH_3_^+^) as ligand. The ionized amantadine was prepared in BIOVIA program by adding one hydrogen ion to the unionized amandatine structure. The redocking experiments were performed with the substrate/substrate analog with which the AKs were co-crystallized in the PDB; the 3D structure of the ligands was downloaded from the PDB Database as sdf file and visualize it on Dassault Systèmes BIOVA program – Discovery Studio Modeling Environment, Release 2017, San Diego: Dassault Systèmes, 2016 (http://accelrys.com). Further preparations for molecular docking were done by AutoDockTools.

#### Molecular docking protocol

Protein-ligand docking experiments were performed with the AutoDock4.2 program, distributed as open source under a GPL license (http://autodock.scripps.edu) [53–55]. The molecular docking data analysis was made on Windows platform using the Dassault Systèmes BIOVA program. Also, Cygwin DLL (cygwin1.dll) terminal was used for the running of molecular docking scripts on Windows platform. The molecular docking algorithm employed was with a rigid AKs structure and a ligand flexibility with 1-6 number of torsion and aromaticity criterion at 7.5’. The searching parameter was the Lamarckian Genetic Algorithm (LGA), with a maximum of 2,500,000 energy evaluations. Other docking parameters were used as defaults.

### Multiple sequence alignment

Multiple sequence alignment was performed by Clustal Omega program (https://www.ebi.ac.uk/Tools/msa/clustalo).

## Results and Discussion

### The molecular docking of AKs with amantadine ionized form (with –NH_3_^+^ group) and the redocking experiments with substrates or analog substrates

There are nine human AKs isoforms described until now, but only those isoforms with X-ray 3D structure solved were further analyzed by molecular docking. The results of human AKs were compared with AKs from other organisms. The AKs with 3D structure co-crystallized with substrates or analog substrates are optimal for our study because the AK is in closed-conformation, with the flexible LID domain covering the substrate. Because the study was designed based on the AKs in closed-conformation, the redocking experiments of AKs with its substrate or analog substrate were performed. The energy binding (Δ*G*) and the inhibition constant (*K*_i_) of the best conformation of the complex AK-amantadine and the complex AK-ligand after redocking allowed a first view of the interaction of amantadine with AKs (Table 1).

#### Redoking of the AKs isoforms with the substrates co-crystallized in the X-ray 3D structures

**Figure 1.**
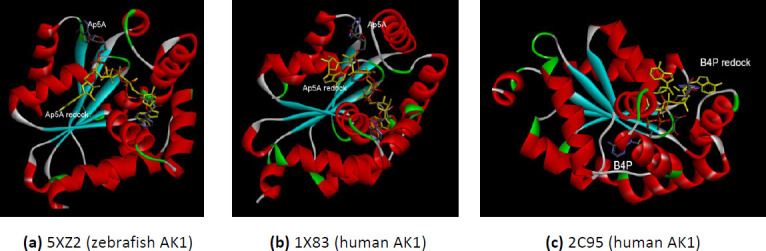
The comparative interactions of the AK1 isoforms with the analog substrates (Ap5A or B4P) after redocking. The redocked conformations are shown in yellow. **(a)** zebrafish AK1 (PDB ID 5XZ2); **(b)** human AK1 (PDB ID 1X83); **(c)** human AK1 (PDB ID 2C95).

The redocking process allows verifying the docking parameters. Because the AK undergoes conformational changes during catalysis, the substrate does not perfectly fit with the PDB conformation on redocking experiments. One explanation is that the crystal structure was solved by an analog substrate, not by the AMP and ATP. The flexibility of the AK is very well studied. During the phosphoryl transfer, the two small peripheral domains of the AK (NMB binding domain and LID domain) move to close the ATP binding region. The AMP is tightly bound to the AMP site when the AMP domain undergoes minor movements. Contrary, when the ATP loosely bounds to the ATP site, the large movements of the LID domain occurs – ~30 °C and 88° hinge bending rotation – then the LID domain closed tightly upon ATP [56]. However, observing the redocked conformations permits the chosen of the most suitable 3D structure when two closed-conformation of the same enzyme, co-crystallized with different substrates or analog substrates, was found in PDB. This is the case of AK isoform 1 for which there are three closed-conformation structures – two for human enzyme and one for zebrafish counterpart. The best redocked conformations are for the AKs co-crystallized with bis(adenosine)-5’-pentaphosphate (Ap5A) – 1X83 and 5XZ2 (Figure 1). The availability of three different conformation of the AK1 – an AK isoform dysregulated in PD – provides a substantial advantage for further analyses.

**Figure 2.**
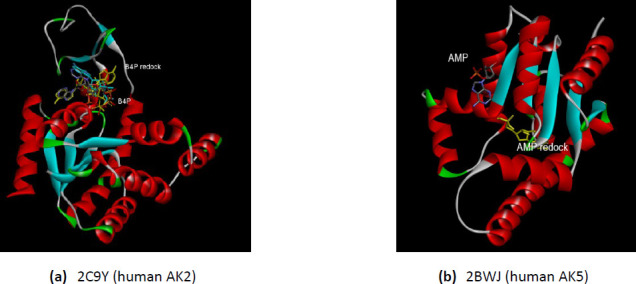
**(a)** The interactions comparative of the human AK2 (PDB ID 2C9Y) with the B4P after redocking; **(b)** The comparative interactions of the human AK5 (PDB ID 2BWJ) with the AMP after redocking. The redocked conformations are shown in yellow.

The human AK2 isoform (PDB ID 2C9Y) is found in closed-conformation only co-crystallized with P1, P4-Di(adenosine)tetraphosphate (B4P). The redocking of the B4P substrate as ligand demonstrate that the parameters specified in the docking method are reasonable even there is not a perfect match with the X-ray 3D structure from the PDB (Figure 2a). The human AK5 isoform (PDB ID 2BWJ) is deposed in PDB only co-crystallized with one substrate – AMP. Therefore, the enzyme is in semi closed-conformation. The redocking of the AMP does not show the same interactions as the X-ray 3D structure deposed in PDB (Figure 2b). The explanation could reside in the semi closed-conformation of the 2BWJ – the 3D structure contains only one substrate AMP, the substrate that causes minor movements of the AMP domain [56]. The semi-closed conformation of the human AK5 is one limitation of the present study, which relies on the X-ray 3D structure on closed-conformation.

**Figure 3.**
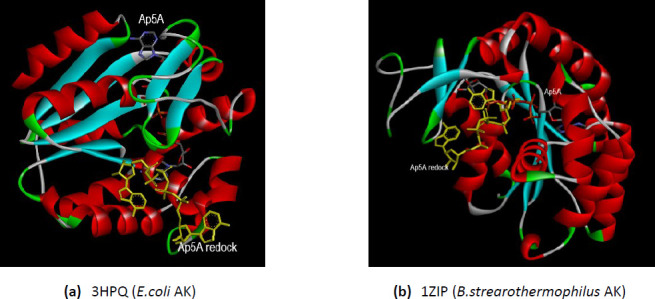
The comparative interactions of the bacterial AKs with the analog substrate Ap5A after redocking. The redocked conformations are shown in yellow. (**a**) *E.coli* AK (PDB ID 3HPQ); (**b**) *B.stearothermophilus* AK (PDB ID 1ZIP).

The redocking of the analog substrate Ap5A as a ligand with AKs from *E.coli* and *B.stearothermophilus* does not perfectly match with the conformation of the Ap5A from the X-ray 3D structure deposed in PDB (Figure 3). Despite this limitation, the closed-conformation of AK is the best choice when performing molecular studies involving this enzyme. Furthermore, the present study’s results allow the comparison of the interaction of the human AKs and bacterial AKs with amantadine.

The AK4 is also named AK3-like and is a GTP:ATP phosphotransferase (EC 2.7.4.10) [57–59]. The mitochondrial human AK4 (PDB ID 2BBW) was deposed on PDB in closed-conformation co-crystallized with diguanosine-pentaphosphate (GP5). The AK4 has some characteristics that differentiate it from other AK isoforms, the most significant being its catalytic activity (a ribonucleoside 5'-diphosphate + ATP = a ribonucleoside 5'-triphosphate + ADP) (https://www.uniprot.org/uniprot/P27144). Because the parameters in the input file do not allow substrate redocking, the molecular docking of amantadine with AK4 was not made.

### Molecular docking of ionized amantadine with AK1

**Figure 4.**
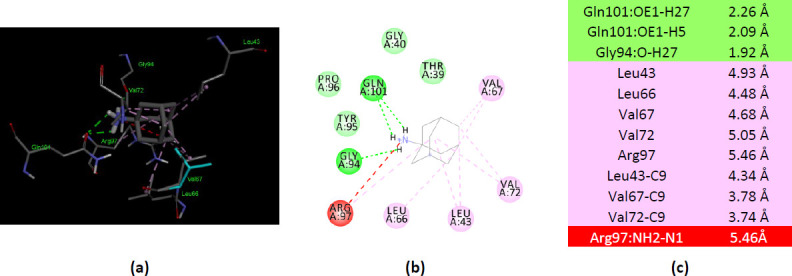
Interactions of ionized amantadine with human AK1, PDB ID 1Z83. (**a**) 3D display of ionized amantadine interaction as ligand with the 1Z83 residues; (**b**) code color for interactions: in green are shown conventional hydrogen bonds, light green – van der Waals forces, mauve – alkyl hydrophobic interactions, red – unfavorable positive-positive interactions; (**c**) the distances (Å) of the 1Z83 - ionized amantadine interactions.

The three X-ray 3D structures of the AK1 were analyzed for their interaction with ionized amantadine (with –NH_3_^+^ group). The human AK1 co-crystallized with Ap5A (PDB ID 1Z83) mostly interacts with ionized amantadine with the residues involved in AMP binding. Thus, the enzyme forms van der Waals forces with Thr39, Tyr95, and Pro96; conventional hydrogen bonds with Gly94 and Gln101; and an unfavorable positive-positive interaction with Arg97. In total, there are five residues from the NMP binding region that interact with ionized amantadine – Thr39, Gly40, Leu43, Leu66, and Val67 (Figure 4). A comparison of the AK1 (PDB ID 1Z83) interactions with the two forms of amantadine, reveals that the group –NH_3_^+^ of the ionized amantadine is involved in three conventional hydrogen bonds like the –NH_2_ group of the unionized form. The difference lies in the residues involved in these interactions – Val67 interacts with –NH_2_ of unionized amantadine, respectively Gln94 and Gln101 with –NH_3_^+^ of ionized amantadine.

**Figure 5.**
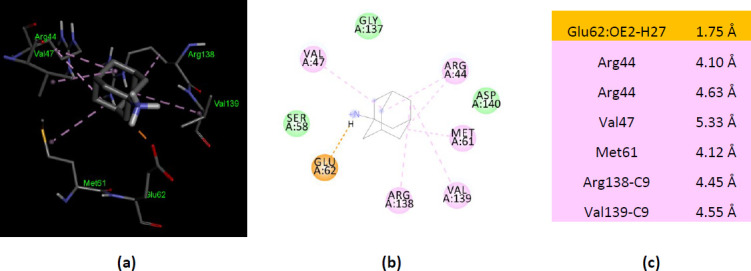
Interactions of ionized amantadine with human AK1, PDB ID 2C95. (**a**) 3D display of ionized amantadine interaction as ligand with the 2C95 residues; (**b**) code color for interactions: in orange are shown the salt bridge, light green – van der Waals forces, mauve – alkyl hydrophobic interactions; (**c**) the distances (Å) of the 2C95 - ionized amantadine interactions.

The human AK1 co-crystallized with B4P (PDB ID 2C95) forms an electrostatic interaction between Glu62, a residue belonging to NMP binding region, and –NH_3_^+^ group of ionized amantadine. The same residue, Glu62, interacts with the –NH_2_ group of the unionized amantadine by two conventional hydrogen bonds. In contrast with human AK1 (PDB ID 1Z83), the human AK1 (PDB ID 2C95) interacts with ionized amantadine with residues belonging to NMP binding region (Arg44, Val47, Ser58, Met61, and Glu62) and LID domain (Gly137, Arg138, Val139, and Asp140). More, AK1 (PDB ID 2C95) interacts with only two AMP binding residues – Arg44 and Arg138 (Figure 5).

**Figure 6.**
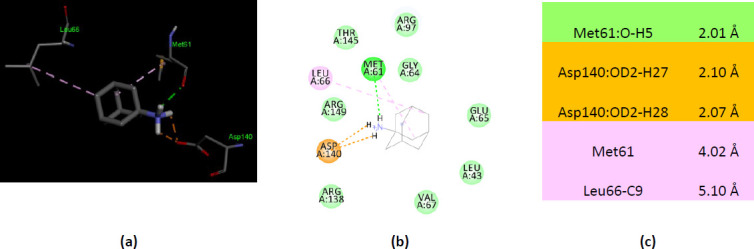
Interactions of ionized amantadine with zebrafish AK1, PDB ID 5XZ2. (**a**) 3D display of ionized amantadine interaction as ligand with the 5XZ2 residues; (**b**) code color for interactions: in green are shown conventional hydrogen bonds, orange – salt bridge, light green – van der Waals forces, mauve – alkyl hydrophobic interactions; (**c**) the distances (Å) of the 5XZ2 - ionized amantadine interactions.

The zebrafish AK1 co-crystallized with Ap5A (PDB ID 5XZ2) shows much more differences regarding the interactions with ionized amantadine, no residue which interacts with unionized amantadine interact with ionized amantadine. In contrast with unionized amantadine, the –NH_3_^+^ group of the ionized amantadine interacts with the residues involved in catalysis – one residue from the NMP binding region, Met61, forms a conventional hydrogen bond and Asp140 from the LID domain forms two electrostatic interactions. Similar to human AK1 (PDB ID 2C95), the zebrafish AK1 interacts with ionized amantadine by residues from the NMP binding region (Leu43, Met61, Gly64, Glu65, Leu66, and Val67) and with residues from the LID region (Arg138 and Asp140). Most of the residues that interact with ionized amantadine are AMP binding residues – Gly64, Glu65, Leu66, Val67, Arg 138, and Arg 149 (Figure 6).

### Molecular docking of ionized amantadine with human AK2

**Figure 7.**
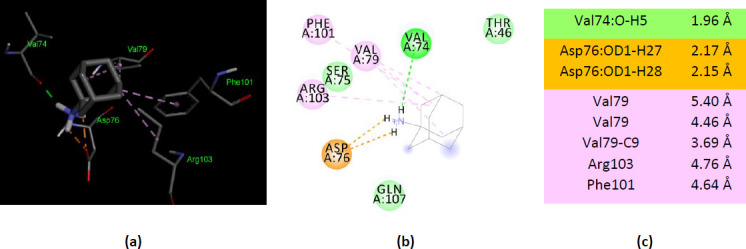
Interactions of ionized amantadine with human AK2, PDB ID 2C9Y. **(a)** 3D display of ionized amantadine interaction as ligand with the 2C9Y residues; **(b)** code color for interactions: in green are shown conventional hydrogen bonds, orange – salt bridge, light green – van der Waals forces, mauve – alkyl hydrophobic interactions; **(c)** the distances (Å) of the 2C9Y-ionized amantadine interactions.

Human AK2 (PDB ID 2C9Y) shows different interactions with the two forms of amantadine. The –NH_3_^+^ group forms a conventional hydrogen bond with a residue from the AMP binding region (Val74) and two electrostatic interactions with Asp76. Two residues from the AMP binding region form alkyl interactions (Arg103 and Phe101). Unlike unionized amantadine, the ionized amantadine shows a better match with the residues from the catalytic site. Ionized amantadine interacts with five AMP binding residues – Thr44, Val74, Phe101, Arg103, and Gln107 (Figure 7).

### Molecular docking of ionized amantadine with human AK5

**Figure 8.**
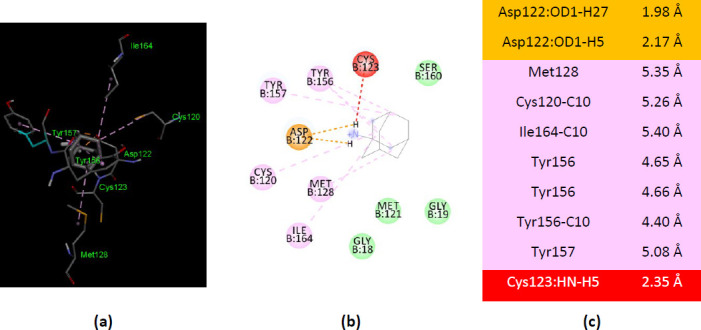
Interactions of amantadine with human AK5, PDB ID 2BWJ. (**a**) 3D display of ionized amantadine interaction as ligand with the 2BWJ residues; (**b**) code color for interactions: in orange are shown salt bridge, light green – van der Waals forces, mauve – alkyl hydrophobic interactions, red – unfavorable donor-donor interactions; (**c**) the distances (Å) of the 2BWJ - ionized amantadine interactions.

Unlike unionized amantadine, the ionized amantadine interacts with human AK5 (PDB ID 2BWJ-chain B) residues that do not belong to the domains involved in catalysis. The van der Waals forces are observed with two glycines in the phosphate-binding loop (P-loop) (Figure 8).

### Molecular docking of ionized amantadine with bacterial AKs

**Figure 9.**
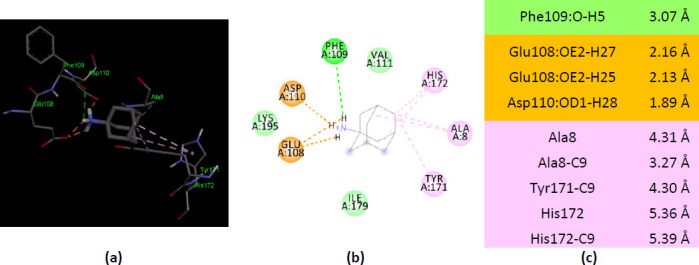
Interactions of ionized amantadine with AK from *E.coli*, PDB ID 3HPQ. (**a**) 3D display of amantadine interaction as ligand with the 3HPQ residues; (**b**) code color for interactions: in green are shown conventional hydrogen bonds, orange – salt bridge, light green – van der Waals forces, mauve – alkyl hydrophobic interactions; (**c**) the distances (Å) of the 3HPQ - ionized amantadine interactions.

A comparison of human AKs with their bacterial counterparts was necessary to draw a valid conclusion about the specificity of human AKs for amantadine. Bacterial AKs with X-ray 3D structure deposited in PDB are much numerous than AKs from eucaryotes. AK from *E.coli* (PDB ID 3HPQ) interacts with ionized amantadine through residues that do not belong to the catalytic site. Only Ala8 (from the P-loop) forms two alkyl hydrophobic interactions with ionized amantadine (Figure 9).

**Figure 10.**
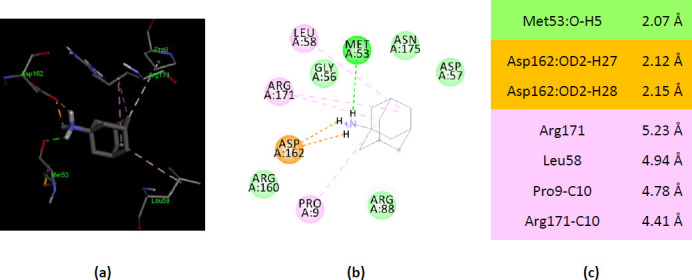
Interactions of ionized amantadine with AK from *B.stearothermophilus*, PDB ID 1ZIP. (**a**) 3D display of amantadine interaction as ligand with the 1ZIP residues; (**b**) code color for interactions: in green are shown conventional hydrogen bonds, orange – salt bridge, light green – van der Waals forces, mauve – alkyl hydrophobic interactions; (**c**) the distances (Å) of the 1ZIP - ionized amantadine interactions.

AK from *B. stearothermophilus* (PDB ID ZIP) interacts with ionized amantadine from two residues belonging to the LID region – with Arg162 by two electrostatic interactions and with Arg160 by van der Waals forces. The Pro9 from the P-loop forms alkyl interactions with ionized amantadine. Also, four residues from the NMP binding region interact with ionized amantadine – Met53, Leu58, Gly56, and Arg171. More, five AMP-binding residues interact with ionized amantadine – Gly56, Asp57, Leu58, Arg88, and Arg171 (Figure 10).

### Comparison of molecular docking of ionized amantadine with the AKs studied

**Figure 11.**
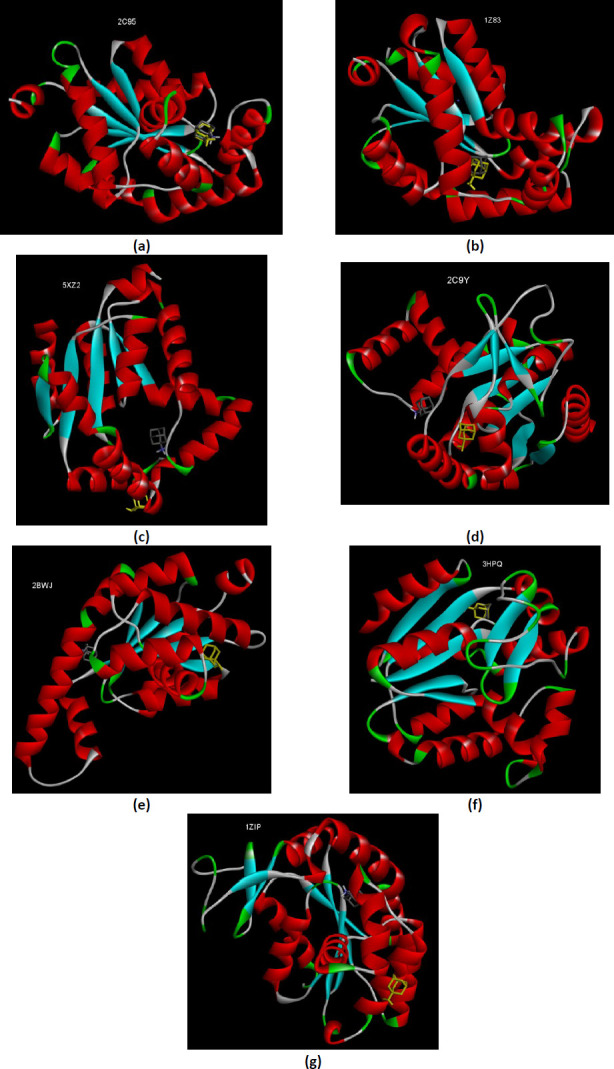
Interactions of unionized and ionized amantadine with AKs. (**a**) human AK1 (PDB ID 2C95); (**b**) human AK1 (PDB ID 1Z85) (**c**) zebrafish AK1 (PDB ID 5XZ2); (**d**) human AK2 (PDB ID 2C9Y); (**e**) human AK5 (PDB ID 2BWJ) (**f**) AK from *E.coli* (PDB ID 3HPQ); (**g**) AK from *B.stearothermophilus* (PDB ID 1ZIP); unionized amantadine is shown in yellow.

The overall comparison of the molecular docking results offers a better view of the interactions of the two forms of amantadine with the human and bacterial AKs. As the physiological form of amantadine is ionized, the comparison of molecular docking results with both forms of the drug has advanced the study on the hypothesis of the AK – amantadine – PD connection. Among the AKs included in the present study the zebrafish AK1 (PDB ID 5XZ2), human AK2 (PDB ID 2C9Y), human AK5 (PDB ID 2BWJ), and AK from *B.stearothermophilus* (PDB ID 1ZIP) show the most differences in interactions with the two forms of amantadine (Figure 11).

### Multiple sequence alignment of the AKs isoforms

The multiple sequence alignment allows an overview of the interactions of the two forms of amantadine with the AKs isoforms. The residues involved substrate binding are well conserved among bacterial AKs and eucaryotes AKs. The interaction of unionized amantadine with zebrafish AK1 and human AK4 mainly involves the residues from the LID region that interact with unionized amantadine. In contrast, the human AK1, and AK5 interact with unionized amantadine through AMP binding residues. Minor similarities are noticed between human AK2 and AK from *E.coli*, both of which have two residues in the phosphate-binding loop (P-loop) that interact with unionized amantadine – one residue glycine being identical for the two enzymes. Also, human AK1 shows some similarities with human AK4 with three different residues from the LID region.

**Figure 12.**
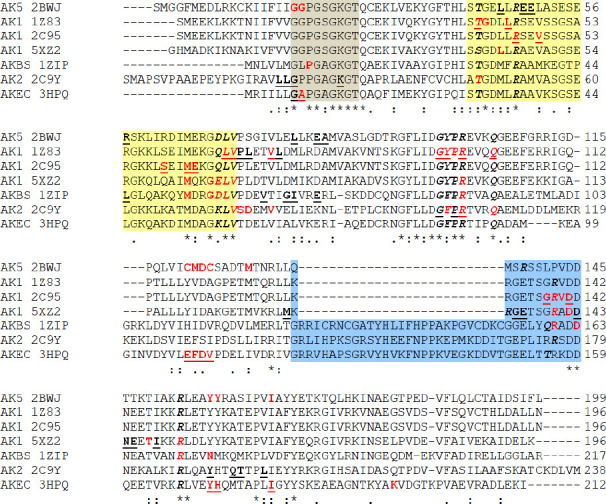
Multiple sequence alignment of the AKs sequences. In gray background are marked the residues belong to Walker A motif – phosphate-binding loop or P-loop; in yellow background is marked the NMP binding region and the residues that belong to AMP binding region are italic-bolded; in blue background is marked the LID region; with red are marked the residues (van der Waals, conventional hydrogen bond or alkyl interactions) which interact with amantadine ionized form (with –NH_3_^+^ group); the residues which interact with amantadine unionized form (with –NH_2_ group) are bolded-underlined; the residues which interact with amantadine unionized and ionized are red and bolded-underlined; AKEC is AK from *E.coli*; AKBS is AK from *B.stearothermophilus*; with dot “.” are marked the semi-conservative replacements; with colon “:” are marked the conservative replacements; with “*” are marked the identities of the residues.

An in-depth analysis of the two forms of ionized amantadine shows that, except for AK5 (PDB ID 2BWJ), the ionized amantadine interacts predominantly with AMP binding residues. Among AK1 isoforms, the interactions with ionized amantadine are not similar. The AK2 (PDB ID 2C9Y) and the *B.stearothermophilus* AK (PDB ID 1ZIP) show a better match with the residues at the catalytic site with the ionized amantadine. Unlike unionized amantadine, the ionized amantadine interacts with more AMP binding residues of AK1 and AK2 isoforms (Figure 12).

### Molecular docking of the human adenine phosphoribosyltransferase with ionized amantadine and the redocking with the substrates

The human APRTs (E.C. 2.4.2.7) found in PDB were analyzed for the amantadine interactions. The enzyme catalyses the reaction: AMP + diphosphate = 5-phospho-alpha-D-ribose 1-diphosphate + adenine. In the adenine metabolism, APRT is involved in the first stage of the subpathway that synthesizes AMP from adenine (UniProtKB P07741). Thus, in adenine metabolism, APRT is closely connected with AK by the substrate AMP. Therefore, the APRT was the first enzyme tested to verify the hypothesis that amantadine specifically binds some human AKs isoforms. First, the redock of the substrates co-crystallized in the X-ray 3D structures was made. Then, amantadine ionized form was tested and the interactions with the residues from the catalytic site (Table2).

**Figure 13.**
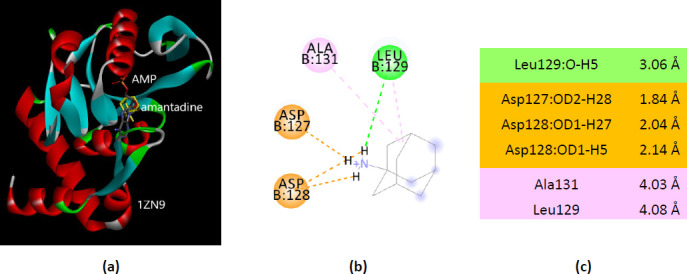
Interactions of ionized amantadine with APRT co-crystallized with AMP, PDB ID 1ZN9. (**a**) 3D display of the amantadine (shows in yellow) and AMP in binding pocket; (**b**) code color for interactions: in green are shown conventional hydrogen bonds, orange – salt bridge, mauve – alkyl hydrophobic interactions; (**c**) the distances (Å) of the 1ZN9 - ionized amantadine interactions.

Among the four PDB structure co-crystallized with adenosine monophosphate (AMP) (PDB ID(s) 1ORE, 1ZN8, 1ZN9, and 4X44), two of them show the best interaction of amantadine with the AMP-binding residues. The amantadine – 1ZN9 interactions involve four residues that also bind AMP (Figure 13).

**Figure 14.**
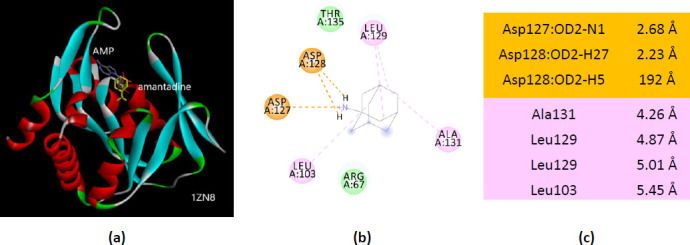
Interactions of ionized amantadine with APRT co-crystallized with AMP, PDB ID 1ZN8. (**a**) 3D display of the amantadine (shows in yellow) and AMP in binding pocket; (**b**) code color for interactions: in orange are shown the salt bridges, light green – van der Waals forces, mauve – alkyl hydrophobic interactions; (**c**) the distances (Å) of the 1ZN8 - ionized amantadine interactions.

Similarly, amantadine – 1ZN8 interactions involve seven AMP-binding residues. Among them, four residues are the same observed in amantadine – 1ZN9 interactions (Asp127, Asp128, Leu129, and Ala131) (Figure 14).

**Figure 15.**
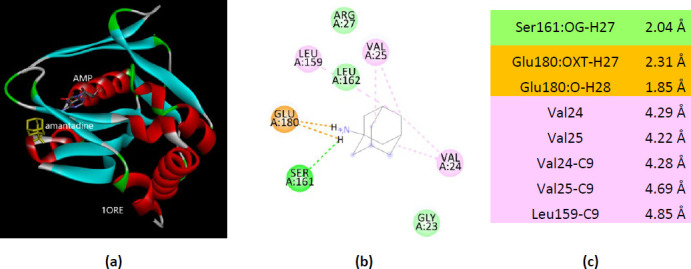
Interactions of ionized amantadine with APRT co-crystallized with AMP, PDB ID 1ORE. (**a**) 3D display of the amantadine (shows in yellow) and AMP in binding pocket; (**b**) code color for interactions: in orange are shown the salt bridges, light green – van der Waals forces, mauve – alkyl hydrophobic interactions; (**c**) the distances (Å) of the 1ORE - ionized amantadine interactions.

The other two APRT structures co-crystallized with AMP show different interactions. Among the eight residues of 1ORE that interact with amantadine only two are AMP-binding residues (Val25 and Arg27) (Figure 15).

**Figure 16.**
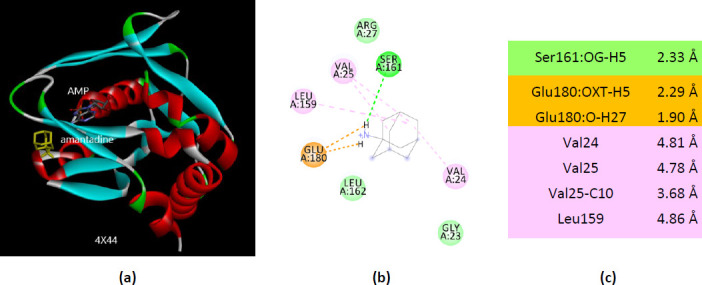
Interactions of ionized amantadine with APRT co-crystallized with AMP, PDB ID 4X44. (**a**) 3D display of the amantadine (shows in yellow) and AMP in binding pocket; (**b**) code color for interactions: in orange are shown the salt bridges, light green – van der Waals forces, mauve – alkyl hydrophobic interactions; (**c**) the distances (Å) of the 4X44 - ionized amantadine interactions.

The molecular docking of amantadine with 4X44 results in similar interactions with 1ORE. There are three AMP-binding residues that bind amantadine (Val25, Arg27, and Leu159) (Figure 16).

**Figure 17.**
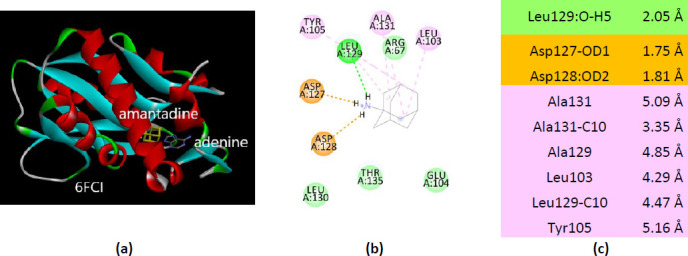
Interactions of ionized amantadine with APRT co-crystallized with adenine, PDB ID 6FCI. (**a**) 3D display of the amantadine (shows in yellow) and adenine in binding pocket; (**b**) code color for interactions: in orange are shown the salt bridges, light green – van der Waals forces, mauve – alkyl hydrophobic interactions; (**c**) the distances (Å) of the 6FCI - ionized amantadine interactions.

The human APRT co-crystallized with adenine (PDB ID(s) 6FCI and 1ZN7) allows a deeper view of the amantadine interactions with this enzyme. The two structures have different behavior when docked with amantadine. Thus, among ten residues of the 6FCI that interact with amantadine, five bind adenine (Glu104, Arg67, Leu129, Tyr105, and Ala131) (Figure 17).

**Figure 18.**
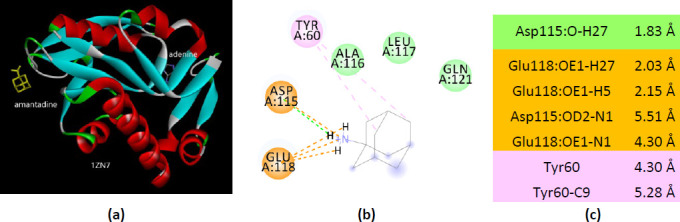
Interactions of ionized amantadine with APRT co-crystallized with adenine, PDB ID 1ZN7. (**a**) 3D display of the amantadine (shows in yellow) and adenine in binding pocket; (**b**) code color for interactions: in orange are shown the salt bridges, light green – van der Waals forces, mauve – alkyl hydrophobic interactions; (**c**) the distances (Å) of the 1ZN7 - ionized amantadine interactions.

Contrary, in the case of 1ZN7 structure, molecular docking of amantadine involves different residues than adenine (Figure 18). The last two structures (PDB ID(s) 6FCI and 1ZN7), being co-crystallized with adenine, not with the substrate AMP, should be interpreted accordingly.

### Molecular docking of the human ecto-5’-nucleotidase (NT5E) with ionized amantadine and the redocking with the substrates

The ecto-5’-nucleotidase (NT5E) (E.C. 3.1.3.5) catalyzes the hydrolysis of extracellular AMP to adenosine. All human NT5Es found in PDB were analyzed about the amantadine interactions. First, the redock of the substrates co-crystallized in the X-ray 3D structures was made, then amantadine ionized form molecular docking (Table 3). In all NT5Es structures analyzed, the amanatdine binds other residues than the substrate-binding region (data not shown).

### Molecular docking of the human ectonucleoside triphosphate diphosphohydrolase 1 with ionized amantadine and the redocking with the substrates

The ectonucleoside triphosphate diphosphohydrolase 1 (ENTPD1) (E.C. 3.6.1.5) catalyzes the reaction: a ribonucleoside 5’-triphosphate + 2H_2_O = a ribonucleoside 5’-triphosphate +2H^+^ + 2phosphate. Because there are no matching PDB entries for human ENTDP1, were analyzed the ENTDP1 from *Rattus norvegicus*. Although the interpretation of the molecular docking of amantadine is not reliable, because the crystal structures are not with the substrates, it is worth noticing that the molecular docking of the 3ZX2 with amantadine results in an interaction with the active-site residue Glu174 by two salt bridge (Table 4).

### Molecular docking of the human nucleoside diphosphate kinase 3 (NDK3) with ionized amantadine and the redocking with the substrates

The nucleoside diphosphate kinase 3 (NDK3) (E.C. 2.7.4.6) catalysis the reaction: a 2’-deoxyribonucleoside 5’-diphosphate + ATP = a 2’-deoxyribonucleoside 5’-triphosphate + ADP. The human found in PDB were analyzed about the amantadine interactions (Table 5). First, the redock of the substrates co-crystallized in the X-ray 3D structures were made for three structures (PDB ID(s) 1ZS6, 2HVD, and 3BBB). In the case of 1ZS6 and 3BBB, no significant interactions of the amantadine with ATP-binding residues or with the histidine from the active site were observed. The 2HVD and 1JXV form van der Waal forces with histidine 118 from the active site (Pros-phosphohistidine intermediate) but no interactions with the ATP-binding residues.

### Molecular docking of the human purine nucleoside phosphorylase 1 (PNP1) with ionized amantadine and the redocking with the substrates

The human purine nucleoside phosphorylase 1 (PNP1) (E.C. 2.4.2.1) catalyses the reaction: a purine D-ribonucleotide + phosphate = a purine nucleobase + alpha-D-ribose 1-phosphate. The reaction is part of purine nucleoside salvage (UniProtKB P00491). The human PNP1s retrieved from the PDB were analyzed about the amantadine interactions (Table 6). The molecular docking of the PNP1 structures shows no relevant interactions with amantadine. Some of the PNP1 interact with amantadine with only one binding-site residue – the 1ULB interacts with Asn243 and the 1RFG forms a salt bridge with Glu201. The 2A0W, 2A0X, 2A0Y, 1RSZ form van der Waals interactions with only one binding-site residue - Gln201.

### Molecular docking of the creatine kinases (CKs) with ionized amantadine and redocking

The CK (E.C. 2.7.3.2) catalyses the reversible reaction: ATP + creatine = ADP + H^+^ + N-phosphocreatine. The different CKs type (UniProtKB P11009, P17540, P00563, P06732, P12277, P05122, and Q9XSC6) retieved form the PDB were analysed about the amantadine interactions (Table 7). Molecular docking results show no interactions of amantadine with ATP-binding residues or nucleotide (ATP) - binding regions.

## Discussion

The PD is an idiopathic, progressive disease of the central neurological system. The treatment of dyskinesia remains a critical issue in the PD treatment strategy, levodopa-induced dyskinesia being a real challenge for both patients and healthcare professionals. The combination of levodopa - amantadine is useful for the people who do not tolerate optimal doses of levodopa and allows better control of the disease. Among other biomarkers modified in PD, there is a dysregulation of some human AK isoforms that are also altered in other muscle diseases. The AK1 and AK2 isoforms are also involved in modulation immunity and inflammation in many diseases. These observations formed the basis of the design of this study.

Because AK is an important enzyme for energetic metabolism, its involvement in a variety of diseases is not surprising. Because the ATP is the most used “exchange currency” in nature, the crucial role of AK is energetic metabolism explains the interest in the study of this enzyme. Nine human AKs isoforms have been identified, each with important characteristics that differentiate them [60]. Five AKs isoforms have the X-ray structure deposited in public databases but only four isoforms are in closed-conformation (AK1, AK2, AK4, and AK5). Consequently, only the AKs in closed-conformation were selected for the present study – five human AKs, one AK1 from zebrafish, and two bacterial AKs.

**Figure 19.**
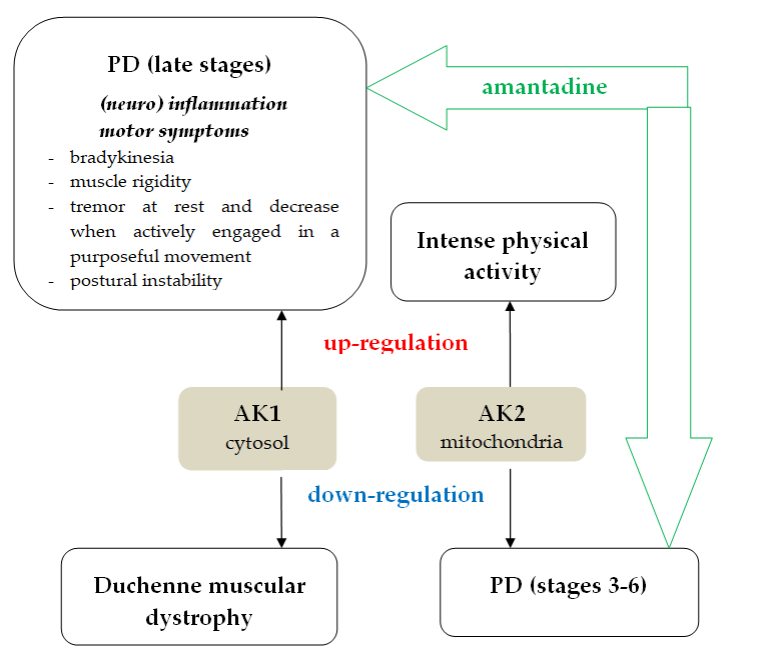
The involvement of human AK1 and human AK2 in PD, inflammation, and muscle activity. The amantadine treatment efficacy in PD developed the hypothesis that amantadine – AK interactions.

Experimental studies are very often costly and time-consuming, so conducting an experimental study without strong theoretical research is not a pragmatic approach. Molecular docking is a useful method for first analyzes of new protein-ligand interaction. This method allowed analysis of the selective binding of amantadine to the AKs’ isoforms implicated in PD. The ΔG and Ki data provide only a first view of the amantadine binding to different AKs. Thus, a deep analysis of the amantadine with the AKs residues illustrated that there are notable differences between all human AKs investigated. It was previously described a down-regulation of the mitochondrial isoforms AK2 and AK4 in the stages 3-6 and an up-regulation of the cytosolic isoform AK1 in the stages 5-6, as compensation of altered purine metabolism (Figure 19) [24]. There are well-known motor deficits in PD – slowness of movement, muscle rigidity, and tremor at rest, the tremor that decreases when the body is actively engaged in purposeful activity [61]– [63]. The cytosolic AK1 and the mitochondrial AK2 are dysregulated in different muscle pathology. In contrast with PD, in Duchenne muscular dystrophy – an X-linked recessive disease – there is a progressive weakness mainly of the pelvic girdle and shoulder girdle, pseudohypertrophy due to the infiltration by fat and connective tissue with immobilization at young ages and cardiomyopathy [64], [65]. The role of AK1 in inflammation has been demonstrated, but its role in maintaining neuroinflammation is not yet known (Figure 19) [66].

According to the scientific literature, there is no connection between the amantadine and AKs in PD. However, because the mechanism of amantadine in PD is not yet known, the investigation of the possible mechanism of amantadine is of great interest. The present study was conducted with the premise that some markers up-regulated or down-regulated in PD could be the target for amantadine. Many studies described the association of some clinical symptoms with dysregulation of different enzymes. The observation that many AKs isoforms are altered in PD leads to the hypothesis that there is a connection between amantadine and these AKs isoforms. Also, other enzymes already described to be dysregulated in PD was investigated – APRT, NT5E, ENTPD1, NDK3, and PNP1 [24]. More, CK was included in the study because of its proven involvement in some muscle diseases [27].

Our experiments show that the human cytosolic AK1 interacts mainly with ionized amantadine by residues from the AMP binding region. Even if the two human AK1s analyzed do not form the same interactions with amantadine, the observation could be aware of the efficacy of the amantadine in the late stages of PD, because, as mentioned earlier, AK1 activity could compensate for purine metabolism dysfunctions. Regarding AK1 from other species, the zebrafish AK1 interactions with amantadine are more similar to those of the AK from *B.stearothermophilus*. Moreover, the interactions with ionized amantadine are different for the three AK1 enzymes analyzed. Regarding the mitochondrial human AK2, the interactions with ionized amantadine are most similar to AK1 (PDB ID 1Z83) with five residues in common – Thr44, Val74, Phe101, Arg103, and Gln107. Thus, the results of the present study highlight the relevance of AK1 and AK2 in PD and provide a valid perspective for further investigation of the role of amantadine in inhibiting these enzymes. The connection between cytosolic and mitochondrial human AKs was extensively studied by Dzeja *et al*. who made a very clear demonstration of the type ”bucket-brigade” process by which AK1 and AK2 isoforms facilitate the transfer of the enzyme substrates without changes in the metabolite concentration in the cellular compartments [67].

It is not possible to establish the mechanism of action of amantadine in PD observing only the interaction of the drug with AKs, but the results of the present study provide a valuable perspective for understanding the pathogenesis of PD. So the next step was to analyze of a large group of proteins. Among the enzymes analyzed for their affinity for amantadine, APRT shows remarkable results. Because there are available many APRTs X-ray 3D structures co-crystallized with AMP and with adenine, we were able to compare the molecular docking results. The molecular docking of amantadine ionized form with APRTs demonstrated that amantadine interacts with AMP-binding residues. Although the structures of APRTs co-crystallized with adenine shows identical residues that bind amantadine and adenine, there are some differences between the APRTs structures tested. Contrary, the results obtained by molecular docking of amantadine with APRTs X-ray 3D structures co-crystallized with AMP strongly indicated that amantadine could target adenine metabolism.

Here, a preliminary analysis of some key factors described in PD pathogenesis reveals that there are at least two enzymes involved in adenine metabolism that worth further investigation – AK and APRT. Then, before an experimental confirmation of the molecular docking results, a molecular dynamics simulation study aid to observe the proper binding of amantadine.

The present study aimed to try to make sense of the anti-parkinsonian effects of the amantadine. The methods used in the present study do not elucidate the mechanism of amantadine, but demonstrate that in PD there is a link between amantadine and some enzymes described to be modified in different stages of the disease.

## Conclusions

The PD is a progressive neurodegenerative disease characterized mainly by motility impairment. There is no curative treatment but there are some therapeutic strategies for the treatment of dyskinesia, amantadine being used until now even for better control of levodopa-induced dyskinesia. Also, there is demonstrated that in the clinical evolution of PD, many enzymes are modified - some AK isoforms, APRT, NT5E, ENTPD1, NDK3, and PNP1. Using available X-ray 3D structures of these enzymes or their counterparts from other organisms, two enzymes show relevant results – AK and APRT. The closed-conformation of the AK is most reliable due to the catalytic particularities of the enzyme – the movement of the domains is coordinated by the sequential addition of the substrates. The molecular docking experiments demonstrated that human AK1 and AK2 with ionized amantadine by AMP binding residues but there are notable differences between the two AK isoforms. Other AKs included in the study, further demonstrated the specific interaction of amantadine with human AKs. The study of amantadine interaction with different AKs’ isoforms has more to give. The studies of the PD highlighted that there is a relationship between the key factors involved in PD. Among them, mitochondrial factors have a crucial role in the disease pathogenesis. The APRT structures co-crystallized with AMP results in the best interactions with amantadine, the AMP-binding residues form strong interactions with amantadine. The results of the present study offer a new perspective for further investigation of the connections between amantadine treatment of PD and some enzymes involved in purine metabolism.

**Table S1.** The characteristics of the AKs included in the study.

	PDB ID	Organism	Residues	co-crystallized with substrate or analog substrate	mutations	reference
AK1	2C95	*Homo sapiens*	194	B4P ^1^; malonate ion	no	unpublished
AK1	1Z83	*Homo sapiens*	196	Ap5A ^2^; SO _4_ ^2-^; Zn ^2+^	no	unpublished
AK1	5XZ2	*Danio rerio*	196	Ap5A; SO _4_ ^2-^	no	[ 32]
AK2	2C9Y	*Homo sapiens*	242	B4P; 1,2-ethandiol	no	unpublished
AK4	2AR7	*Homo sapiens*	246	no	no	unpublished
AK4	2BBW	*Homo sapiens*	246	GP5 ^3^	no	unpublished
AK5	2BWJ	*Homo sapiens*	199	AMP	R135M	unpublished
	3HPQ	*E.coli*	214	Ap5A	no	[ 33]
	1ZIP	*B. stearothermophilus*		Ap5A; Mn ^2+^ ; Zn ^2+^	no	[ 34]

^1^ P1, P4-Di(adenosine)tetraphosphate

^2^ Bis(adenosine)-5’-pentaphosphate

^3^ diguanosine-pentaphosphate.

**Table S2.** The characteristics of the APRTs included in the study.

PDB ID	Organism	Residues	co-crystallized with substrate or analog substrate	mutations	reference
1ORE	*Homo sapiens*	180	AMP ^1^; Cl ^-^	no	[ 35]
1ZN7	*Homo sapiens*	180	PRP ^2^; HSX ^3^, ADE ^4^; PO _4_ ^-^; Mg _2_ ^+^	no	[ 36]
1ZN8	*Homo sapiens*	180	AMP; Cl ^-^	no	[ 36]
1ZN9	*Homo sapiens*	180	AMP	no	[ 36]
4X44	*Homo sapiens*	180	AMP, SO _4-_; GOL ^5^	R89Q	unpublished
6FCI	*Homo sapiens*	180	PRP; ADE; Mg _2_ ^+^	no	[ 37]

^1^ AMP – adenosine monophosphate

^2^alpha-phosphoribosylpyrpphosphoric acid

^3^5-O-phosphono-alpha-D-ribofuranose

^4^adenine

^5^glycerol

**Table S3.** The characteristics of the NT5Es included in the study.

PDB ID	Organism	Residues	co-crystallized with substrate or analog substrate	mutations	reference
4H2I	*Homo sapiens*	532	A12 ^1^; Zn _2_ ^+;^ Ca _2_ ^+;^ Cl ^-^	no	[ 38]
4H2G	*Homo sapiens*	546	AND ^2^, Zn _2_ ^+;^ Ca _2_ ^+;^ Cl ^-^	seven	[ 38]
4H2F	*Homo sapiens*	546	AND ^2^, Zn _2_ ^+;^ Ca _2_ ^+;^ Cl ^-^	seven	[ 38]
4H1S	*Homo sapiens*	530	NAG ^3^; PO _4_ ^-^; Zn _2_ ^+^	no	[ 39]

^1^ phosphomethylphosphonic acid adenosyl ester

^2^ adenosine

^3^ N-actetyl-D-glucosamine

**Table S4.** The characteristics of the NDK3s included in the study.

PDB ID	Organism	Residues	co-crystallized with substrate or analog substrate	mutations	reference
1ZS6	*Homo sapiens*	169	ADP ^1^	no	unpublished
2HVD	*Homo sapiens*	152	ADP	no	[ 40]
2HVE	*Homo sapiens*	152	ADP	S120G	[ 40]
1JXV	*Homo sapiens*	152	no	no	[ 41]
3BBB	*Homo sapiens*	151	DG ^2^; DA ^3^	no	[ 42]

^1^ adenosine-5’-diphosphate

^2^ 2’-deoxyguanosine-5’-monophosphate

^3^ 2’-deoxyadenosine-5’-monophosphate

**Table S5.** The characteristics of the PNP1s included in the study.

PDB ID	Organism	Residues	co-crystallized with substrate or analog substrate	mutations	reference
1ULA	*Homo sapiens*	289	SO _4_ ^-^	no	[ 43]
1ULB	*Homo sapiens*	289	guanine; SO _4_ ^-^	no	[ 43]
2A0W	*Homo sapiens*	289	DIH ^1^; SO _4_ ^-^	H257G	[ 44]
2A0X	*Homo sapiens*	289	DIH ^1^; SO _4_ ^-^	H257F	[ 44]
2A0Y	*Homo sapiens*	289	DIH ^1^; SO _4_ ^-^	H257D	[ 44]
1RSZ	*Homo sapiens*	289	DIH ^1^; SO _4_ ^-^	no	unpublished
1RFG	*Homo sapiens*	288	guanosine; SO _4_ ^-^	no	[ 45]

**^1^** 7-[[(3R,4R)-3-(hydroxymethyl)-4-oxidanyl-pyrrolidin-1-ium-1-yl]methyl]-3,5-dihydropyrrolo[3,2-d]pyrimidin-4-one

**Table S6.** The characteristics of the CKs included in the study.

PDB ID	Residues	Organism	co-crystallized with substrate or analog substrate	mutations	reference
mitochondrial CK					
1CRK	380	*Gallus gallus*	SO _4_ ^-^	no	[ 46]
4Z9M	392	*Homo sapiens*	ADP ^1^	no	unpublished
muscle CK					
2CRK	381	*Oryctolagus cuniculus*	SO _4_ ^-^	no	[ 48]
1I0E	381	*Homo sapiens*	no	no	[ 48]
1U6R	380	*Oryctolagus cuniculus*	ADP; IOM ^2^; NO _3_ ^-^; Mg _2+_	R134K	[ 49]
brain CK					
3DRB	381	*Homo sapiens*	ADP; Mg _2+_	no	[ 50]
3DRE	381	*Homo sapiens*	no	no	[ 50]
1QH4	380	*Gallus gallus*	acetate ion; Ca _2_ ^+^	no	[ 51]
retinal CK					
1G0W	380	*Bos taurus*	SO _4_ ^-^	no	[ 52]

^1^ adenosine-5’-diphosphate

^2^ (diaminomethyl-methyl-amino)-acetic acid

## Figures and Tables

**Table 1.** The energy binding (Δ*G*) and inhibition constant (*K*_i_) of the best conformation of the complex AK - amantadine and after redocking.

AK	PDB ID	Amantadine ionized form		Amantadine unionized form		Redocking^1^	
		Δ*G* (kcal/mol)	*K*_i_	Δ*G* (kcal/mol)	*K*_i_	Δ*G* (kcal/mol)	*K*_i_
Human cytosolic AKs							
AK1	2C95	-6.19	29.04 μM	-6.47	18.1 μM	-10.95	9.36 nM
AK2	1Z83	-5.97	42.33 μM	-6.05	36.94 μM	-11.14	6.87 nM
AK5	2BWJ	-6.79	12.61 μM	-7.03	7.03 μM	-4.46	534.4 μM
Human mitochondrial AKs							
AK2	2C2Y	-6.00	39.68 μM	-6.25	26.29 μM	-7.38	3.91 μM
AK4	2BBW	Not performed				Fail	
Zebrafish AK							
AK1	5XZ2	-5.51	91.53 μM	-5.69	67.9 μM	-13.2	209.44 pM
Bacterial AKs							
*E.coli*	3HOQ	-6.55	15.78 μM	-6.38	21.21 μM	-5.62	75.52 μM
*B.stearothermophilus*	1ZIP	-5.87	49.41 μM	-5.77	59.42 μM	-7.2	5.3 μM

^1^Redocking with the substrates co-crystallized in PDB X-ray 3D structures.

**Table 2.** The energy binding (Δ*G*) and inhibition constant (*K*_i_) of the best conformation of the complex APRT - amantadine (ionized form) and after redocking.

PDB ID	Amantadine			redocking^1^	
	Δ*G* (kcal/mol)	*K*_i_ (μM)		Δ*G* (kcal/mol)	*K*_i_ (μM)
1ORE	-6.44		18.93	-6.91	8.68
1ZN9	-6.52		16.77	-6.3	24.04
4X44	-6.01		39.53	-8.1	1.15
1ZN8	-6.15		31.21	-7.39	3.83
6FCI	-7.33		4.26	-6.21	28.14
1ZN7	-6.9		8.73	-5.58	81.74

^1^ redocking with the substrates co-crystallized in PDB X-ray 3D structures.

**Table 3.** The energy binding (ΔG) and inhibition constant (Ki) of the best conformation of the complex NT5E - amantadine and after redocking.

PDB ID	Amantadine			redocking^1^	
	Δ*G* (kcal/mol)	*K*_i_ (μM)		Δ*G* (kcal/mol)	*K*_i_ (μM)
4H2I	-8.08		1.2	-4.91	250.92
4H2F	-7.93		1.55	-6.95	8.01
4H2G	-7.59		2.72	-5.97	42.08
4H1S	-7.12		6.08	-5.56	84.56

^1^ redocking with the substrates co-crystallized in PDB X-ray 3D structures.

**Table 4.** The energy binding (ΔG) and inhibition constant (Ki) of the best conformation of the complex ENTPD1 - amantadine.

PDB ID	Amantadine			redocking	
	Δ*G* (kcal/mol)	*K*_i_ (μM)		
3ZX0	-6.6		14.56	Not the case	
3ZX2	-6.45		18.71		
3ZX3	-7.24		4.94		

**Table 5.** The energy binding (ΔG) and inhibition constant (Ki) of the best conformation of the complex NDK3 - amantadine and after redocking.

PDB ID	Amantadine			redocking^1^	
	Δ*G* (kcal/mol)	*K*_i_ (μM)		Δ*G* (kcal/mol)	*K*_i_ (μM)
1ZS6	-7.34		4.15	-5.96	42.42
2HVD	-6.55		15.69	-5.81	54.99
1JXV	-7.13		5.97	not the case	
3BBB	-5.63		74.71	-5.88^2^ -5.35^3^	49.34^2^ 119.96^3^

^1^ redocking with the substrates co-crystallized in PDB X-ray 3D structures

^2^ redock of 2’-deoxyadenosine-5’-monophosphate

^3^ redock of 2’-deoxyguanosine-5’-monophosphate.

**Table 6.** The energy binding (ΔG) and inhibition constant (Ki) of the best conformation of the complex PNP1 - amantadine and after redocking.

PDB ID	Amantadine			redocking^1^	
	Δ*G* (kcal/mol)	*K*_i_ (μM)		Δ*G* (Kcal/mol)	*K*_i_
1ULA	-7.31		4.35	not the case	
1ULB	-6.74		11.47	-5.61	77.85 μM
2A0W	-6.52		16.57	-8.63	473.78 nM
2A0X	-6.5		17.29	-8.69	425.71 nM
2A0Y	-6.46		18.38	-8.89	306.03 nM
1RSZ	-6.41		19.92	-8.35	760.14 nM
1RFG	-6.4		20.45	-7.42	3.67 μM

^1^ redocking with the substrates co-crystallized in PDB X-ray 3D structures.

**Table 7.** The energy binding (ΔG) and inhibition constant (Ki) of the best conformation of the complex CK - amantadine and after redocking.

PDB ID	Amantadine			redocking^1^		
	Δ*G* (kcal/mol)	*K*_i_		Δ*G* (Kcal/mol)	*K*_i_	
mitochondrial CK						
1CRK	-6.65		13.26 μM	not the case		
4Z9M	-7.3		4.49 μM	-8.64		462.56 nM
muscle CK						
2CRK	-7.65		2.45 μM	not the case		
1IOE	-5.81		54.93 μM	not the case		
1U6R	-6.37		21.24 μM	-7.84		1.78 μM
brain CK						
3DRB	-6.39		20.63 μM	not the case		
3DRE	-6.99		7.57 μM	not the case		
1QH4	-7.45		3.44 μM	not the case		
retinal CK						
1G0W	-7.12		6.07 μM	not the case		

^1^ redocking with the substrates co-crystallized in PDB X-ray 3D structures.
